# p53/sirtuin 1/NF-κB Signaling Axis in Chronic Inflammation and Maladaptive Kidney Repair After Cisplatin Nephrotoxicity

**DOI:** 10.3389/fimmu.2022.925738

**Published:** 2022-07-07

**Authors:** Ying Fu, Ying Wang, Yuxue Liu, Chengyuan Tang, Juan Cai, Guochun Chen, Zheng Dong

**Affiliations:** ^1^ Department of Nephrology, Hunan Key Laboratory of Kidney Disease and Blood Purification, The Second Xiangya Hospital at Central South University, Changsha, China; ^2^ Department of Cellular Biology and Anatomy, Medical College of Georgia at Augusta University, Augusta, GA, United States; ^3^ Charlie Norwood Veterans Affairs (VA) Medical Center, Augusta, GA, United States

**Keywords:** cisplatin, inflammation, kidney injury and repair, NF-κB, renal fibrosis, sirtuin 1

## Abstract

Chronic inflammation contributes to maladaptive kidney repair, but its regulation is unclear. Here, we report that sirtuin 1 (SIRT1) is downregulated after repeated low-dose cisplatin (RLDC) injury, and this downregulation leads to p65 acetylation and consequent NF-κB activation resulting in a persistent inflammatory response. RLDC induced the down-regulation of SIRT1 and activation of NF-κB, which were accompanied by chronic tubular damage, tubulointerstitial inflammation, and fibrosis in mice. Inhibition of NF-κB suppressed the production of pro-inflammatory cytokines and fibrotic phenotypes in RLDC-treated renal tubular cells. SIRT1 activation by its agonists markedly reduced the acetylation of p65 (a key component of NF-κB), resulting in the attenuation of the inflammatory and fibrotic responses. Conversely, knockdown of SIRT1 exacerbated these cellular changes. At the upstream, p53 was activated after RLDC treatment to repress SIRT1, resulting in p65 acetylation, NF-κB activation and transcription of inflammatory cytokines. In mice, SIRT1 agonists attenuated RLDC-induced chronic inflammation, tissue damage, and renal fibrosis. Together, these results unveil the p53/SIRT1/NF-κB signaling axis in maladaptive kidney repair following RLDC treatment, where p53 represses SIRT1 to increase p65 acetylation for NF-κB activation, leading to chronic renal inflammation.

## Introduction

Nephrotoxicity is the major limiting factor for the clinical use and efficacy of cisplatin, a widely used chemotherapy drug for cancer patients (1–4). A single high dose of cisplatin induces acute kidney injury (AKI), which involves renal tubular cell injury and death (5–7). In addition, it induces a robust inflammatory response in kidneys, including the infiltration of immune cells and the production of various pro-inflammatory cytokines (5, 8, 9). In current clinical settings, cisplatin is often administered in multiple cycles at low doses (10), which lead to chronic kidney problems in a significant portion of cancer patients (11). In animals, repeated low dose cisplatin (RLDC) treatment leads to chronic renal pathologies characterized by tubular degeneration, renal inflammation, and interstitial fibrosis, which are associated with a progressive decline of renal function (12–17). However, the mechanism of RLDC-induced chronic renal pathologies remains unclear.

Following AKI, some tubular cells are injured and die, but the surviving cells have the capacity to regenerate and repair the damaged renal tubules. Complete repair can restore the integrity and function of renal tubules for a full recovery. However, after severe AKI or repeated episodes of injury, kidney repair is often incomplete or maladaptive, resulting in atrophic tubules that produce various factors to stimulate inflammation, vascular dropout, myofibroblast expansion and matrix deposition for renal fibrosis (18–21). RLDC is expected to induce maladaptive kidney repair for its repeated toxicity to renal tubular cells. Indeed, following RLDC treatment, mice showed tubular degeneration, chronic inflammation, and varied degrees of interstitial fibrosis depending on the models (12–16). Remarkably, the pathological changes were associated with a progressive decline of renal function (15, 16).

It is generally understood that renal inflammation persists in, and contributes to, maladaptive kidney repair; but the mechanism of chronic renal inflammation during maladaptive kidney repair remains elusive (18–22). Nuclear factor-κB (NF-κB) is a family of “master” transcription factors that are responsible for immune and inflammatory gene expression (23). This family consists of five structurally related members, including NF-κB1 (also called p50), NF-κB2 (also called p52), RelA (also called p65), RelB, and c-Rel, which form hetero- or homo-dimers, i.e. NF-κB. In unstimulated cells, NF-κB is sequestered in the cytoplasm by its inhibitory proteins IκBs (24). Upon stimulation, IκBs is phosphorylated by the IκB kinase (IKK) with consequent ubiquitination and proteasomal degradation (23). Following IκB degradation, NF-κB is released and translocated to the nucleus to induce target gene transcription (25). In the nucleus, p65 (a critical component of the NF-κB complex) is acetylated and the acetylation status of specific lysine residues in p65 affects both the DNA-binding ability and transcriptional activity of NF-κB for various pro-inflammatory cytokines, such as Tnf-α, Il-8, Il-6, and Il-1β (24, 26). NF-κB plays a critical role in renal inflammation in kidney diseases (27). However, the role and regulation of NF-κB in maladaptive kidney repair remains largely unclear.

Mammalian sirtuin 1 (SIRT1) is a protein deacetylase of the highly conserved nicotinamide adenine dinucleotide (NAD^+^) - dependent sirtuin family that is ubiquitously expressed in mammalian cells (28). In addition to its bona fide function of histone deacetylation, SIRT1 also deacetylates lysine residues of various proteins to participate in the aging process (29), inflammation (30), cancer (31), metabolic (32) and neurodegenerative diseases (33). In the kidney, SIRT1 and other sirtuins play important roles in renal function and physiology, and their dysregulation contribute to the pathogenesis of kidney diseases (34). For example, activation of SIRT1 reduced the acetylation of Smad3 and ameliorated fibrosis in 5/6 nephrectomy rats probably by suppressing TGF-β/Smad3 signaling (35). In unilateral ureteral obstruction (UUO), SIRT1 was shown to be an important protective factor for renal medullary interstitial cells against oxidative stress partly by regulating of COX2 (36). In diabetic models, SIRT1 prevent renal fibrosis by suppressing HIF1α, GLUT1, and SNAIL (37). However, the role and regulation of SIRT1 in maladaptive kidney repair remain unclear.

In the present study, we demonstrate that SIRT1 is repressed by p53 during maladaptive kidney repair following cisplatin nephrotoxicity, and the down-regulation of SIRT1 contributes to p65 acetylation and consequent NF-κB activation, resulting in renal inflammation and fibrosis. Therapeutically, targeting the p53/SIRT1/NF-κB signaling axis may improve kidney repair and recovery.

## Methods

### Animal Models

Male C57BL/6 mice (8 weeks) were purchased from SJA Laboratory Animal Corporation (Hunan, China) and maintained in the animal facility of the Second Xiangya Hospital. Mice received saline vehicle or 8 mg/kg cisplatin (Hansoh Pharma, Jiangsu, China) intraperitoneally once a week for 4 weeks (15). For intervention, SRT1720 (a small molecule activator of SIRT1 purchased from APExBIO, Houston, USA) was administered to mice at 100 mg/kg/day (38) by intraperitoneal injection for 7 days after the last cisplatin dose. To inhibit p53, pifithrin-α (APExBIO, Houston, USA) was administered to mice at 2.2 mg/kg/day by intraperitoneal injection for 7 days after the last cisplatin dose. Animals were sacrificed at 4 and 8 weeks after the last cisplatin dose. All animal experiments were performed according to a protocol approved by the Institutional Committee for the Care and Use of Laboratory Animals of the Second Xiangya Hospital at Central South University.

### Cell Culture and Treatments

The Boston University mouse proximal tubular cell line (BUMPT) was originally obtained from Dr. Lieberthal (Boston University) and cultured as previously (15). The cells were subjected to four cycles of treatment with 0, 0.5, 1, or 2 μM cisplatin (15). Each cycle consisted of 7 h of cisplatin incubation and 17 h of recovery in cisplatin-free medium. For intervention, BUMPT cells were initially subjected to 4 cycles of cisplatin treatment. After the final dose, cells were incubated with 100μM TPCA1, 20μM RSV, 2.5μM SRT1720, or 25μM pifithrin-α (all purchased from APExBIO, Houston, USA) for 17h in cisplatin-free medium. Small interference RNA (siRNA) specific against the mouse SIRT1 gene, p53 gene and scrambled control siRNA were synthesized by RiboBio (Guangzhou, China). Cells were transfected with 50 nM Sirt1 siRNA, Trp53 siRNA or control siRNA for 24h using riboFECT_TM_ CP Transfection Kit (RiboBio, Guangzhou, China).

### Transcutaneous Measurement of Glomerular Filtration Rate (GFR)

GFR was measured in mice by transcutaneously monitoring the clearance of FITC-labeled sinistrin as previously (15). Briefly, mice were anesthetized with isoflurane using an anesthesia machine, which contains the breathing circuit, air intake pump, recovery device and induction box. Then, a transdermal GFR monitor (MediBeacon, Mannheim, Germany) was attached to hair-shaved skin of anesthetized mice. FITC-sinistrin (7mg/100g b.w.) was injected through the tail vein. Data analysis is based on the elimination kinetic curve of FITC-sinistrin.

### Histological Analysis

#### Hematoxylin and Eosin Staining

Kidney tissues were fixed with 4% paraformaldehyde, embedded in paraffin, and sectioned at 4 μm for H&E staining. The morphological changes were determined blindly. Kidney cortex and outer medulla were examined to grade tubular damage: absent (0), mild (1), moderate (2), severe (3), and very severe injury (4).

#### Masson Trichrome Staining

Kidney sections were stained using the kit from Servicebio (Wuhan, China). For quantification, 10 positive collagen-stained fields (100 magnification) were randomly selected from each section and analyzed by Image-Pro Plus 6.0 to assess the percentage of fibrotic area.

#### Immunohistochemical Staining

Immunohistochemical staining was performed as previously (15). Primary antibodies tested included those for SIRT1 (8469; Cell Signaling Technology), p65 (8242; Cell Signaling Technology), F4/80 (GB11027, Servicebio, Wuhan, China), and Ki67 (GB111141, Servicebio, Wuhan, China). For quantification, randomly selected 10 fields from each tissue section were evaluated to determine the percentage of positive staining cells per square millimeter.

#### Immunofluorescence

Immunofluorescence staining was conducted as previously (39). After fixation with cold methanol: acetone (1:1), the cells were incubated with a blocking solution containing 10% goat serum, followed by overnight incubation with primary antibodies, and finally with secondary antibodies at 37°C for 1 hour. Primary antibodies for immunofluorescence included those for p65 (8242, Cell Signaling Technology), Collagen I (AF7001, Affinity), p-p65 (3033, Cell Signaling Technology), SIRT1 (8469, Cell Signaling Technology). Cy3-labeled goat anti-rabbit IgG and FITC-labeled goat anti-rabbit IgG were used as secondary antibodies. The cells were counter-stained with Hoechst to show the nucleus and examined on a Zeiss LSM780 confocal microscope (Oberkochen, Germany).

### Extraction of Nuclear and Cytoplasmic Proteins

The extraction was done with a kit was purchased from Beyotime (Shanghai, China). Briefly, cells were centrifuged to collect the cell pellet, which was mixed with PMSF-containing cytoplasmic protein extraction reagent A, vortexed vigorously and then incubated in ice bath. Cytoplasmic protein extraction reagent B was then added. After vortexing, the mixture was centrifuged at 12000g for 5 minutes at 4°C to collect the supernatant as the cytoplasmic fraction. Then, the pellet was re-suspended in the nucleoprotein extraction reagent by vigorous vortexing (15-30s every 1-2 minutes for a total of 30 minutes), and then centrifuged at 12000g for 10 minutes at 4°C to collect the supernatant as the nuclear proteins.

### Immunoblot Analysis

Kidney cortex and outer medullary tissue was lysed in 2% SDS buffer with 1% protease inhibitor cocktail (P8340, Sigma-Aldrich). The samples were resolved on SDS-polyacrylamide gel, and then transferred for immunoblotting using a standard protocol as previously described (40). For densitometry, the protein bands were analyzed with the NIH ImageJ software. The primary antibodies used for immunoblotting included: anti-Fibronectin (ab2413), anti-IL-6 (ab229381) and anti-Tnf-α (ab183218) from Abcam; anti-SIRT1 (8469), anti-NF-κB p65 (8242), anti-phospho-NF-κB p65 (3033), anti-acetyl-NF-κB p65 (Lys310) (3045), anti-GAPDH (5174), anti-Vimentin (5741), anti-phospho-p53 (Ser15) (9284), anti-p53 (2524), anti-Il-1β (12242) and anti-phospho-Histone H2AX (80312) from Cell Signalling Technology; anti-Collagen type I (AF7001) from Affinity. All secondary antibodies for immunoblot analysis were from Thermo Fisher Scientific.

### Quantitative Real-Time PCR

Total RNA was extracted with TRIzol reagents (CWBIO, Jiangsu, China) according to the manufacturer’s protocol. cDNA was synthesized using the PrimeScript RT reagent Kit (TaKaRa, Japan). Quantitative real-time PCR was performed using the TB Green Premix Ex Taq II reagent (TaKaRa, Japan) on a LightCycler96 Real-Time PCR System. Relative expression was normalized to the expression levels of GAPDH. The primer sequences for PCR are listed in **Table 1**.

**Table 1:** Primer sequences used for quantitative RT-PCR.

Gene	Forward	Reverse
***Il-1β***	*5′- GAAATGCCACCTTTTGACAGTG-3′*	*5′- CTGGATGCTCTCATCAGGACA-3′*
***Il-6***	*5′-TCCAGTTGCCTTCTTGGGAC-3′*	*5′-GTACTCCAGAAGACCAGAGG-3′*
***Il-8***	*5′- CTAGGCATCTTCGTCCGTCC -3′*	*5′- TTCACCCATGGAGCATCAGG -3′*
***Tnf-α***	*5′-CAGGCGGTGCCTATGTCTC-3′*	*5′-CGATCACCCCGAAGTTCAGTAG-3′*
***Sirt1***	*5′-CCACTCTCCCTTCTGTCCTCTCTC-3*	*5′-GACCCGCCCTATCTGCTCTCTG-3′*
***Gapdh***	*5′-AGGTCGGTGTGAACGGATTTG-3′*	*5′-GGGGTCGTTGATGGCAACA-3*′

### ChIP Assay

ChIP assay was performed with the ChIP-IT^®^ High Sensitivity kit (Active Motif, USA) according to the manufacturer’s instruction. Briefly, cells were cross-linked with 1% formaldehyde followed by incubation in glycine stop-fix solution. Then, the cells were lysed with the lysis buffer containing protease inhibitor cocktail and deacetylase inhibitors. Purified chromatin was sonicated and incubated with an indicated immunoprecipitation antibody anti-p53 antibody (Cell Signalling Technology, 2524). The resultant immunoprecipitate was subjected to qPCR amplification of putative p53 binding sequences using specifically designed primers. The value of qPCR was normalized with input DNA for comparison. The following primers were used for p53 binding site detection: forward GCGCCAGAGGCCGCTGA and reverse CGGCTGCGGGAGATTTAAACC.

### Statistics

Statistical analysis was performed using the GraphPad Prism software. Multiple group comparison was analyzed with ANOVA followed by Tukey’s posttests. Statistical differences between two groups were determined by two-tailed unpaired or paired Student’s t-test. The value of *P* < 0.05 was considered significantly different.

## Results

### RLDC Treatment Induces Chronic Kidney Injury and Activates NF-κB During Maladaptive Kidney Repair

**Figure 1:**
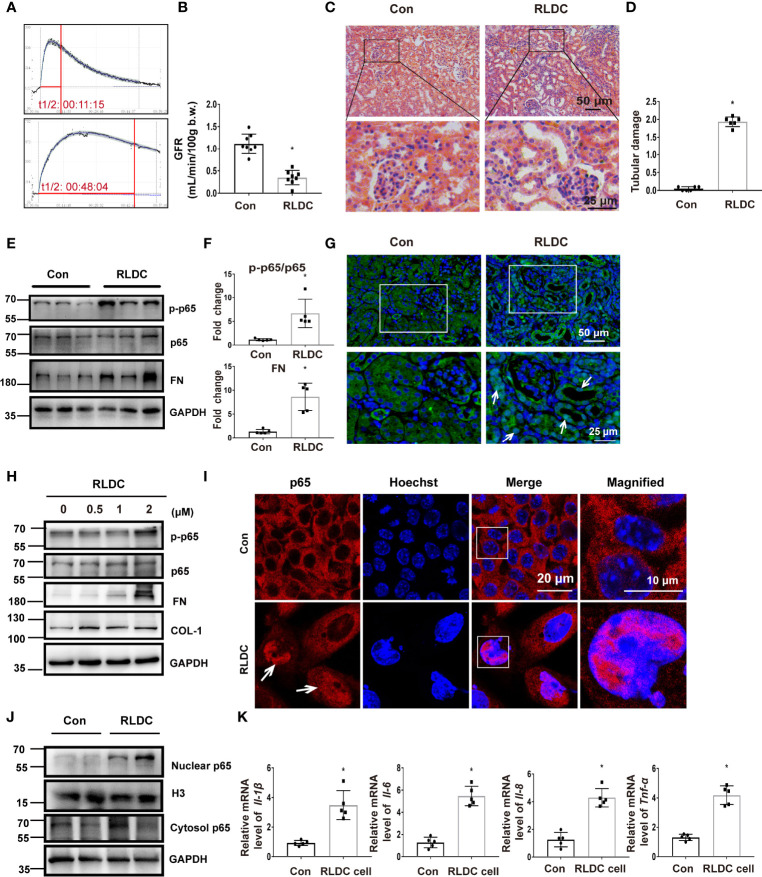
RLDC treatment induces chronic kidney injury and activates NF-κB during maladaptive kidney repair. **(A–G)** Male C57BL/6 mice were injected weekly with 8 mg/kg cisplatin for 4 weeks to collect samples 1 month later. **(A)** The elimination profile of transdermal FITC-sinistrin was monitored. **(B)** GFR measurement by monitoring transcutaneous FITC-sinistrin clearance (n=8). **(C)** Representative histology images of hematoxylin-eosin staining of kidney tissues (n=6). **(D)** Pathological score of tubular damage. **(E)** Representative immunoblots of p-p65, p65, FN, and GAPDH (loading control) in kidney tissues (n=5). **(F)** Densitometry of p-p65, p65 and FN. **(G)** Immunofluorescence analysis of p65 (n=5). Right two images were magnified from the boxed areas. **(H–K)** BUMPT cells were incubated with indicated concentrations of cisplatin for 7h each day for 4 days, and collected at day 5. **(H)** Representative immunoblots of p-p65, p65, FN, COL-1 and GAPDH (loading control) in BUMPT cells (n=5). **(I)** Immunofluorescence analysis of nuclear translocation of NF-κB. Images were collected by laser-scanned confocal microscopy (n=5). Right two images were magnified from the boxed areas. **(J)** Immunoblot of nuclear and cytosolic p65 in RLDC-treated cells (n=5). H3 and GAPDH were used as internal controls for nuclear fractions and whole cell lysate, respectively. **(K)** BUMPT cells were incubated with 2μM cisplatin for 7h each day for 4 days, and collected at day 5. mRNA levels of *Il-1β, Il-6, Il-8, Tnf-α* in cells after RLDC treatment (n=5). Data are normalized to GAPDH and expressed as fold change compared to controls. Data are expressed as mean ± SEM. *P<0.05 *vs*. the control group (Con).

One month after RLDC treatment, mice had a significant decline of renal function as measured by the clearance rate of FITC-labeled sinistrin and the calculated GFR (**Figures 1A, B**). These mice showed clear signs of renal tubular damage, including renal tubule expansion, slight loss of brush border, cast formation and increased inflammatory cell infiltration (**Figure 1C**). Tubular damage was further semi-quantified in cortex and outer medulla (**Figure 1D**). Along with tubular injury was a chronic inflammatory response. The expression of multiple pro-inflammatory cytokines, including interleukins (Il-1β, -6, -8) and tumor necrosis factor (TNF)-α, was markedly increased in post-RLDC kidney tissues (**Supplemental Figure 1A**). Moreover, there was a dramatic increase of macrophages shown by F4/80 staining (**Supplemental Figures 1B, C**). These results indicate that the inflammatory response in injured tubule cells may contribute to chronic renal inflammation and maladaptive repair following RLDC treatment.

NF-κB is a classical transcription factor for the expression of inflammatory cytokines. Upon activation, p65, a key component of NF-κB, is phosphorylated and translocated into the nucleus for downstream gene expression. We detected p65 phosphorylation in post-RLDC kidneys, which was accompanied by increases of fibrosis markers like fibronectin (FN) (**Figures 1E, F**). In immunofluorescence, a considerable portion of p65 moved to the nucleus in renal tubule cells after RLDC treatment (**Figure 1G**). For *in vitro* experiments, we recently established a cell model of RLDC treatment in BUMPT cells (15). Consistently, RLDC induced p65 phosphorylation as well as FN and COL-1 in BUMPT cells in a dose-dependent manner (**Figure 1H**). Moreover, immunofluorescence detected nuclear accumulation of p65 in post-RLDC cells (**Figure 1I**). Immunoblot analysis of nuclear and cytosolic fractions demonstrated a notable increase of p65 in cell nucleus, which was accompanied by a slight increase of p65 in the cytosol (**Figure 1J**). Also in this cell model, RLDC also induced the expression of *Il-1β, Il-6, Il-8*, and *Tnf-α* (**Figure 1K**), suggesting that injured tubule cells might be a major resource of pro-inflammatory cytokines after RLDC treatment. These results indicate that RLDC promotes p65 expression and nuclear accumulation for NF-κB activation, accompanied by the induction of a pro-fibrotic phenotype in renal tubular cells.

### TPCA-1 Reduces the Expression of Pro-Inflammatory Cytokines and Fibrotic Phenotypes in RLDC-Treated Renal Tubular Cells

**Figure 2:**
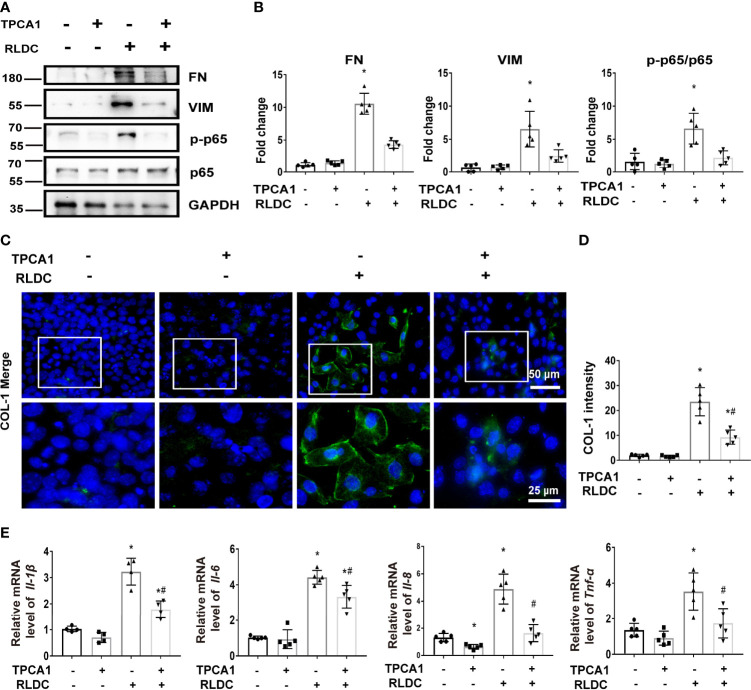
TPCA-1 reduces the expression of pro-inflammatory cytokines and fibrotic phenotypes in RLDC-treated renal tubular cells. BUMPT cells were incubated with 2μM cisplatin for 7h each day for 4 days. After the final dose, RLDC cells were treated with 100μM TPCA1 or saline for 17h to collect samples. **(A)** Representative immunoblots of FN, VIM, p-p65, p65 and GAPDH (loading control) of total cellular proteins after RLDC and TPCA1 treatment (n=5). **(B)** Densitometry of FN, VIM, p-p65, p65. The protein level of control group (TPCA1- RLDC-) was arbitrarily set as 1, and the signals of other conditions were normalized with the control group to indicate their protein fold changes. **(C)** Immunofluorescence analysis of COL-1. (n=5). The images at the bottom are magnified from the boxed area. **(D)** The quantification of COL1-1 intensity. **(E)** mRNA expression of *Il-1β, Il-6, Il-8, Tnf-α* (n=5). Data are normalized to GAPDH and expressed as fold change compared to controls. Data are expressed as mean ± SEM. *P<0.05 *vs*. the control (TPCA1- RLDC-) group, #*P* < 0.05 *vs*. (TPCA1- RLDC+) group.

To determine the role of NF-κB, we evaluated the effect of TPCA-1, which suppresses NF-κB by selectively inhibiting IKK-2 (41, 42). As shown in **Figures 2A, B**, TPCA-1 markedly ameliorated the fibrotic changes in RLDC-treated tubular cells, as indicated by reduced expressions of FN and VIM. Immunofluorescence further verified the inhibitory effect of TPCA-1 on COL-1 expression (**Figures 2C, D**). In addition, TPCA1 attenuated the expression of multiple pro-inflammatory cytokines, including *Il-1β, Il-6, Il-8*, and *Tnf-α* (**Figure 2E**). These data suggest that RLDC triggers aberrant activation of NF-κB in renal tubular cells to stimulate a robust inflammatory response and pro-fibrotic changes.

### RLDC Induces Down-Regulation of SIRT1 and Consequent Acetylation of p65

**Figure 3:**
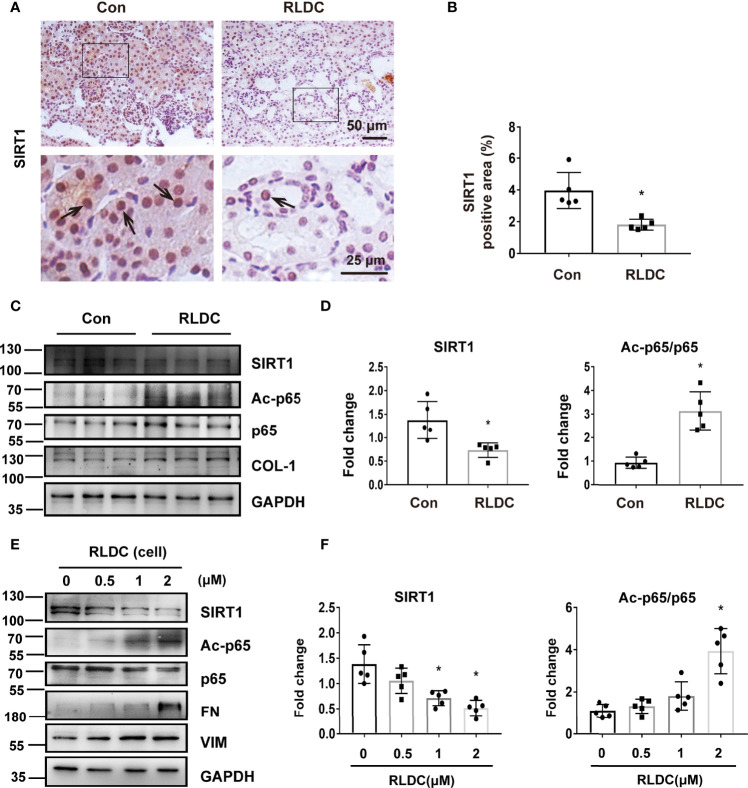
RLDC induces down-regulation of SIRT1 and consequent acetylation of p65. **(A–D)** Male C57BL/6 mice were injected weekly with 8 mg/kg cisplatin for 4 weeks to collect samples 1 month later. **(A)** Representative images of immunohistochemical staining of SIRT1 in mouse kidneys. (n=5). The arrows in the magnified images indicate positive staining. **(B)** Percentage of SIRT1 positive nuclei. **(C)** Representative immunoblots of SIRT1, Ac-p65, p65, COL-1 and GAPDH of total protein extracted from mouse kidney (n=5). GAPDH was used as a loading control. **(D)** Densitometry of SIRT1, Ac-p65, p65. **(E, F)** BUMPT cells were incubated with 2μM cisplatin for 7h each day for 4 days, and collected at day 5. **(E)** Representative immunoblots of SIRT1, Ac-p65, p65, FN, VIM and GAPDH of total protein extracted from BUMPT cells (n=5). GAPDH was used as a loading control. **(F)** Densitometry of SIRT1 and Ac-p65. The protein level of control group was arbitrarily set as 1, and signals of other conditions were normalized with the control group to indicate their protein fold changes. Data are expressed as mean ± SEM. *P<0.05 *vs*. the control group (Con or 0μM).

We then investigated the mechanism of NF-κB activation induced by RLDC. SIRT1 is a powerful deacetylase that has been reported to deacetylate p65 on lysine-310 to inhibit NF-κB (43). Recent work has implicated SIRT1 as a protective factor in cisplatin-induced acute kidney injury (44). We therefore hypothesized that SIRT1 may regulate NF-κB by deacetylating p65 during the development of chronic kidney problems after RLDC treatment. In immunohistochemical staining, SIRT1 was mainly present in the nucleus of tubular cells (**Figure 3A**) and, after RLDC treatment, the SIRT1 signal was decreased to half of control (**Figure 3B**). Importantly, decreased expression of SIRT1 was accompanied by increased Ac-p65 level (**Figures 3C, D**). This expression pattern was further confirmed in BUMPT cells where RLDC markedly decreased SIRT1 expression in a cisplatin dose-dependent manner, while increasing Ac-p65 (**Figures 3E, F**). These results suggest that RLDC may decrease SIRT1 to promote p65 acetylation, contributing to NF-κB activation in post-RLDC kidneys and renal tubular cells.

### SIRT1 Activator Reduces p65/NF-κB Activation and Ameliorates Kidney Inflammation and Fibrosis After Post-RLDC Treatment

Next, we evaluated the therapeutic potentials of SIRT1 agonists in RLDC-treated mice. To this end, after RLDC treatment, the SIRT1 agonist SRT1720 (S) was injected daily into the mice for one week. One group of animals were sacrificed immediately after S treatment to examine its effects on kidney injury, SIRT1 and p65 acetylation, while another group of animals were terminated 3 weeks later to examine the long-term effect of SRT1720 on chronic renal inflammation and interstitial fibrosis (**Supplemental Figure 2A**).

**Figure 4:**
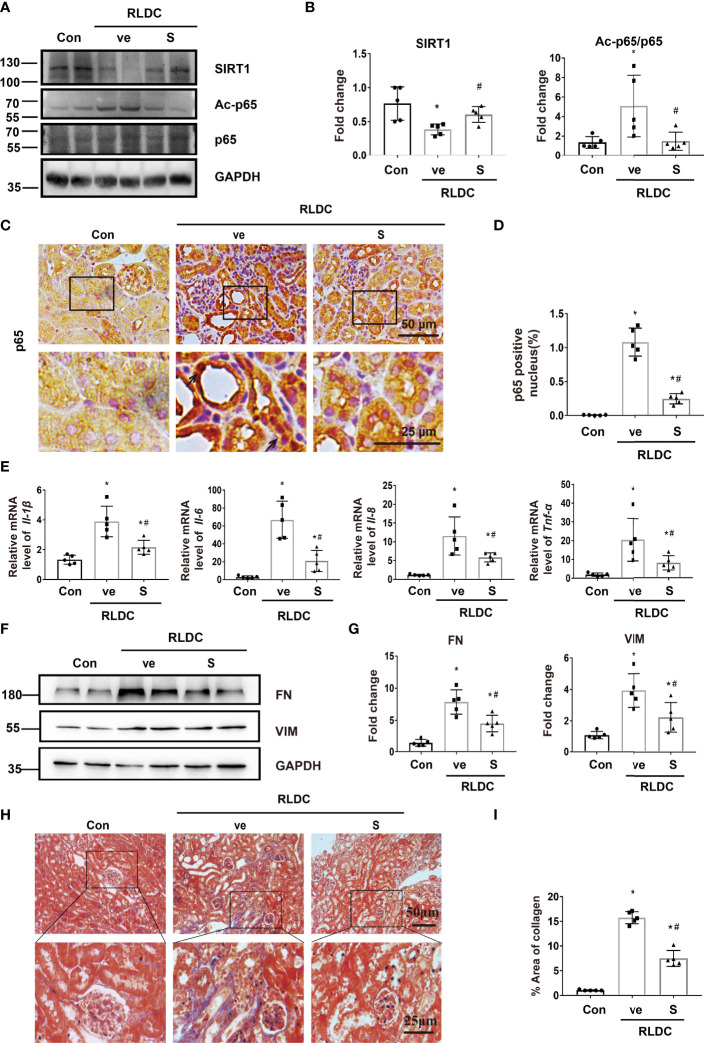
SIRT1 activator reduces p65/NF-κB activation and ameliorates kidney inflammation and fibrosis after post-RLDC treatment. Male C57BL/6 mice were given 4 weekly injections of 8 mg/kg cisplatin. After the last cisplatin injection, SRT1720 (S) or vehicle solution (ve) was injected daily for 1 week or 1 month to collect samples for analysis. **(A)** Representative immunoblots of SIRT1, Ac-p65, p65 and GAPDH (loading control) of total protein extracted from mouse kidney (n=5). **(B)** Densitometry of SIRT1, Ac-p65, p65. The protein level of control group was arbitrarily set as 1. SRT1720 prevented SIRT1 decrease in post-RLDC mice and reduced p65 acetylation. **(C)** Representative images of immunohistochemical staining of p65 in post-RLDC mice. (n=5). **(D)** Percentage of p65 positive nuclei. **(E)** mRNA levels of *Il-1β, Il-6, Il-8, Tnf-α* in mice quantified by qRT-PCR showing the inhibitory effect of SRT1720 (n=5). Data are normalized to *Gapdh* and expressed as fold change compared to controls. **(F)** Representative immunoblots of FN, VIM and GAPDH showing the inhibitory effect of SRT1720 on fibrotic protein expression (n=5). **(G)** Densitometry of FN and VIM. The protein level of control group was arbitrarily set as 1, and the signals of other conditions were normalized with the control group to indicate their protein fold changes. **(H)** Representative images of Masson trichrome staining showing the inhibitory effect of SRT1720. (n=5). **(I)** Quantitative analysis of Masson trichrome staining. Data are expressed as mean ± SEM. *P<0.05 *vs*. the control group (Con + ve), #*P* < 0.05 *vs*. vehicle solution plus RLDC group (RLDC + ve).

In both immunoblot and immunostaining analyses, RLDC induced a marked down-regulation of SIRT1 in kidneys, which was partially but significant prevented by SRT1720. Notably, SRT1720 inhibited p65 activation, manifested by the decrease of Ac-p65 and nuclear p65 (**Figures 4A–D**). Three weeks later, the mice injected with SRT1720 showed reduced inflammatory cytokine production (**Figure 4E** and **Supplemental Figure 3**), and ameliorated renal interstitial fibrosis (**Figures 4F–I**). In addition, SRT1720 reduced kidney atrophy, tubular damage, and renal function decline in post-RLDC kidneys (**Supplemental Figures 2B–F**). Collectively, these findings suggest that activation of SIRT1 may suppress the pro-inflammatory and pro-fibrotic changes in post-RLDC kidneys to improve kidney repair.

To determine how the SIRT1/NF-κB signaling axis affects tubular repair in post-RLDC kidneys, we examined tubular cell proliferation and senescence. We first examined the expression of Ki67, a marker of cell proliferation in renal tubules. A representative image of Ki67 staining and the quantification are shown in **Supplementary Figures 4A, B**. As expected, the control kidney had very few Ki67 positive cells. After RLDC, there was a 2-fold increase in renal tubular Ki67 staining, indicating renal tubular cell proliferation for kidney repair. It is worth noting that SRT1720 (SIRT1 agonist) increased Ki67 staining in renal tubules, indicating that renal repair was enhanced. Premature senescence plays an important role in maladaptive kidney repair including renal fibrosis. In order to explore the influence of SIRT1 activation on premature senescence, we performed SA-β-Gal staining, which is a standard marker of senescence. As shown in **Supplementary Figures 4C, D**, the renal tubules after RLDC treatment showed a significant increase of SA-β-Gal staining, mainly in the renal cortex, which was markedly reduced by SRT1720, suggesting the anti-senescence function of SIRT1 in this model. Furthermore, Ki67- cells with high γ-H2AX lesion density (four or more lesions per nucleus) indicate renal tubular senescence (45). SRT1720 reduced γ-H2AX^+^/Ki67^-^ cells in the kidney (**Supplementary Figures 4E, F**), and reduced the expression of γ-H2AX in immunoblot analysis (**Supplementary Figures 4G, H**). Together, these new results indicate that SIRT1 activation by SRT1720 enhances renal tubular proliferation and ameliorates renal senescence after RLDC, providing further mechanistic insights into the regulation of maladaptive kidney repair by the SIRT1/NF-κB axis.

### SIRT1 Ameliorates Fibrotic and Inflammatory Phenotypes in RLDC-Treated Cells

**Figure 5:**
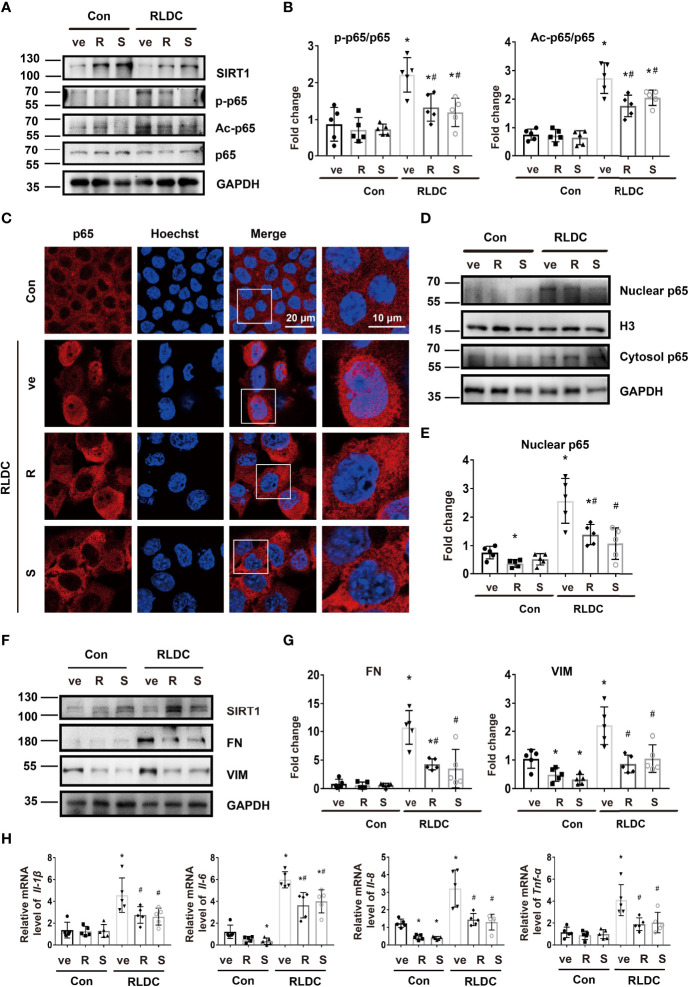
SIRT1 ameliorates fibrotic and inflammatory phenotypes in RLDC-treated cells. BUMPT cells were incubated with 2μM cisplatin for 7h each day for 4 days, and then treated with 20μM resveratrol (R), 2.5μM SRT1720 (S), or vehicle solution (ve) for 17h in cisplatin-free medium to collect samples for analysis. **(A)** Immunoblot of SIRT1, p-p65, Ac-p65 and p65 of total protein extracted from cells (n=5). **(B)** Semi-quantitative analysis of immunoblot of SIRT1, p-p65, and Ac-p65. **(C)** Immunofluorescence analysis of nuclear translocation of p65. Images were collected by laser-scanned confocal microscopy. (n=5). **(D)** Immunoblot analysis of nuclear and cytosolic p65 (n=5). The cells were fractionated into nuclear and cytosolic fractions for immunoblot analysis of p65. H3 and GAPDH were used as internal controls for nuclear and cytosolic fractions, respectively. **(E)** Densitometry of nuclear p65. The protein level of control group was arbitrarily set as 1, and the signals of other conditions were normalized with the control group to indicate their protein fold changes. **(F)** Representative immunoblots of SIRT1, FN, VIM and GAPDH (loading control) of total protein extracted from cells (n=5). **(G)** Densitometry of FN, VIM. The protein level of control group was arbitrarily set as 1, and the signals of other conditions were normalized with the control group to indicate their protein fold changes. **(H)** mRNA levels of *Il-1β, Il-6, Il-8, Tnf-α* were quantified by qRT-PCR (n=5). Data are normalized to *Gapdh* and expressed as fold change compared to controls. *P<0.05 *vs*. the control group (Con + ve), #*P* < 0.05 *vs*. RLDC with vehicle solution group (RLDC + ve).

*In vitro*, resveratrol (R) and SRT1720 (S) could significantly increase SIRT1 expression in both RLDC-treated and control BUMPT cells (**Figure 5A**). Importantly, these SIRT1 activators reduced the expression of Ac-p65 and p-p65 during RLDC treatment, and reduced nuclear accumulation of p65 as shown by immunofluorescence staining (**Figures 5A–C**). Consistently, immunoblot analysis of cellular fractionations showed that SIRT1 activators decreased nuclear p65 whereas increased cytosolic p65 in RLDC-treated cells (**Figures 5D, E**), which indicates that activation of SIRT1 leads to p65 deacetylation resulting in the prevention of p65/NF-κB activation during RLDC treatment. To examine the effects of SIRT1 activators on RLDC-induced maladaptive repair responses, persistent fibrotic changes were induced in BUMPT cells, featured by increased FN and VIM expression. These fibrotic changes were markedly attenuated by SIRT1 activators (**Figures 5F, G**). Moreover, SIRT1 activators reduced the expressions of pro-inflammatory cytokines in RLDC-treated cells, especially *Il-8* and *Tnf-α* (**Figure 5H**). Thus, these findings support that activation of SIRT1 ameliorates the pro-inflammatory and pro-fibrotic changes in RLDC-treated cells.

**Figure 6:**
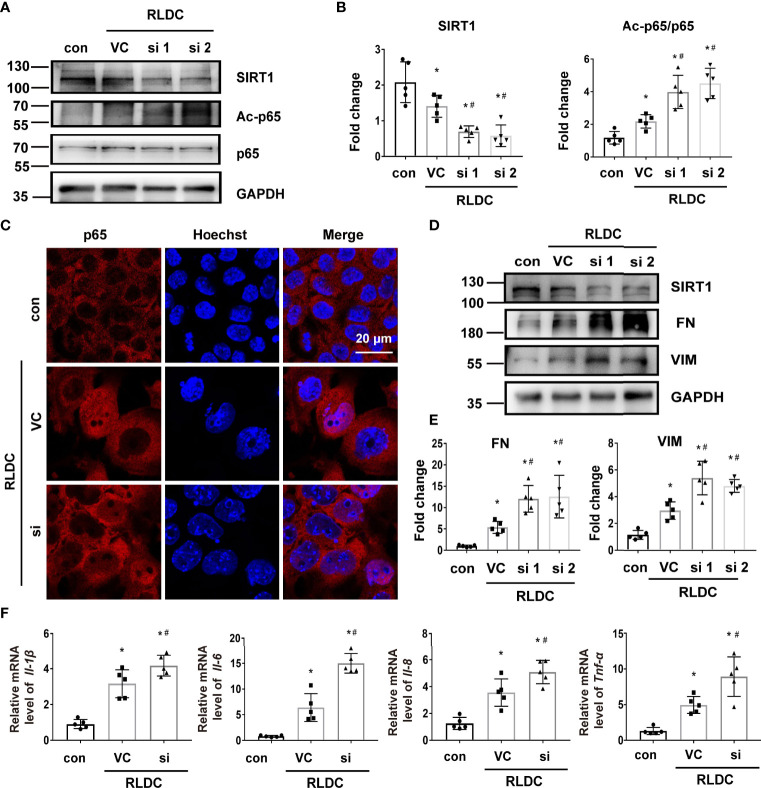
Knockdown of SIRT1 increases p65 acetylation and the fibrotic phenotype of RLDC-treated renal tubular cells. BUMPT cells were transfected with 50nM SIRT1 siRNA1 (si1), siRNA2 (si2), or control siRNA (VC), and then incubated with 2μM cisplatin for 7h each day for 4 days. **(A)** Representative immunoblots of SIRT1, Ac-p65, p65 and GAPDH (loading control) of total protein extracted from cells (n=5). **(B)** Densitometry of SIRT1 and Ac-p65. **(C)** Immunofluorescence analysis of nuclear translocation of p65. (n=5). **(D)** Representative immunoblots of SIRT1, FN, VIM and GAPDH (n=5). **(E)** Densitometry of FN and VIM. The protein level of control group was arbitrarily set as 1, and the signals of other conditions were normalized with it to indicate their protein fold changes. **(F)** mRNA levels of Il-1β, Il-6, Il-8, Tnf-α quantified by qRT-PCR (n=5). Data were normalized to Gapdh and expressed as fold change over controls. Data are expressed as mean ± SEM. *P<0.05 *vs*. the control group (Con), #P < 0.05 *vs*. RLDC with VC group.

We further examined the effects of SIRT1 knockdown. Transfection of SIRT1 siRNAs (si1 and si2) attenuated SIRT1 expression in BUMPT cells. Notably, these cells had 3-4 fold higher expression of Ac-p65 (**Figures 6A, B**), and showed more nuclear accumulation of p65 (**Figure 6C**). Knockdown of SIRT1 aggravated the expression of fibrotic markers (eg. FN and VIM) (**Figures 6D, E**) and pro-inflammatory cytokines (eg. *Il-1β, Il-6, Il-8*, *Tnf-α*) (**Figure 6F**) in RLDC-treated cells. These results further support the conclusion that down-regulation of SIRT1 during maladaptive kidney repair contributes to NF-κB activation, renal inflammation, and fibrosis.

### p53 Mediates the Down-Regulation of SIRT1 After RLDC Treatment

**Figure 7:**
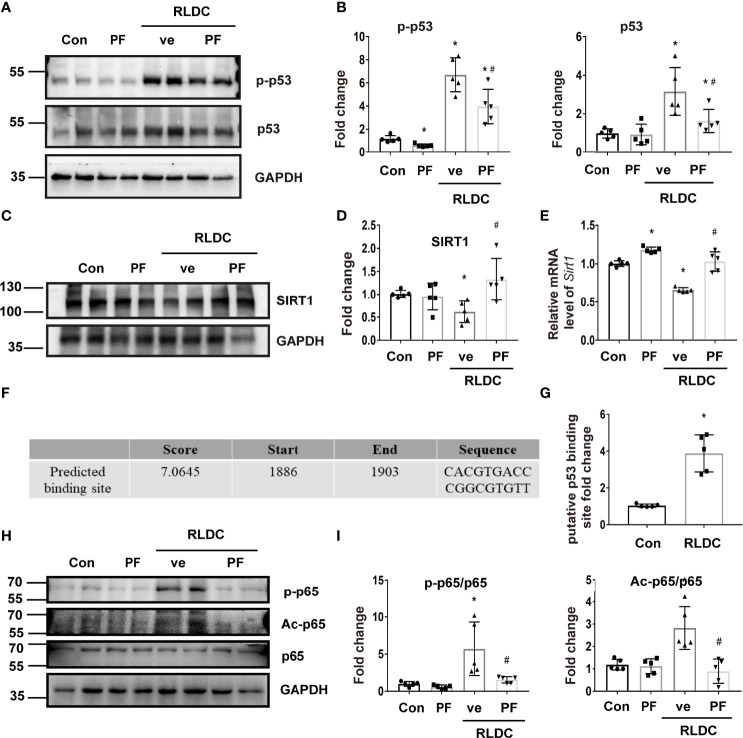
p53 mediates the downregulation of SIRT1 in RLDC-treated renal tubular cells. BUMPT cells were incubated with 2μM cisplatin for 7h each day for 4 days, and then treated with 25μM pifithrin-α (PF) or vehicle solution (ve) for 17h in cisplatin-free medium. **(A)** Representative immunoblots of p-p53, p53 and GAPDH of cell total protein (n=5). **(B)** Densitometry of p-p53 and p53. The protein level of the control group was arbitrarily set as 1, and the signals of other conditions were normalized with the control group to indicate their protein fold changes. **(C)** Representative immunoblots of SIRT1 and GAPDH from total protein (n=5). **(D)** Densitometry of SIRT1. **(E)** Effect of PF on *Sirt1* mRNA expression (n=5). **(F)** p53 binding site on *Sirt1* gene promoter predicted by JASPAR database. **(G)** ChIP assay of p53 binding to SIRT1 promoter sequence after RLDC treatment (n=5). **(H)** Representative immunoblots of p-p65, Ac-p65, p65 and GAPDH from total protein (n=5). **(I)** Densitometry of p-p65 and Ac-p65. Data are expressed as mean ± SEM. *P<0.05 *vs*. the control group (Con), #P < 0.05 *vs*. RLDC with vehicle solution group (RLDC + ve).

**Figure 8:**
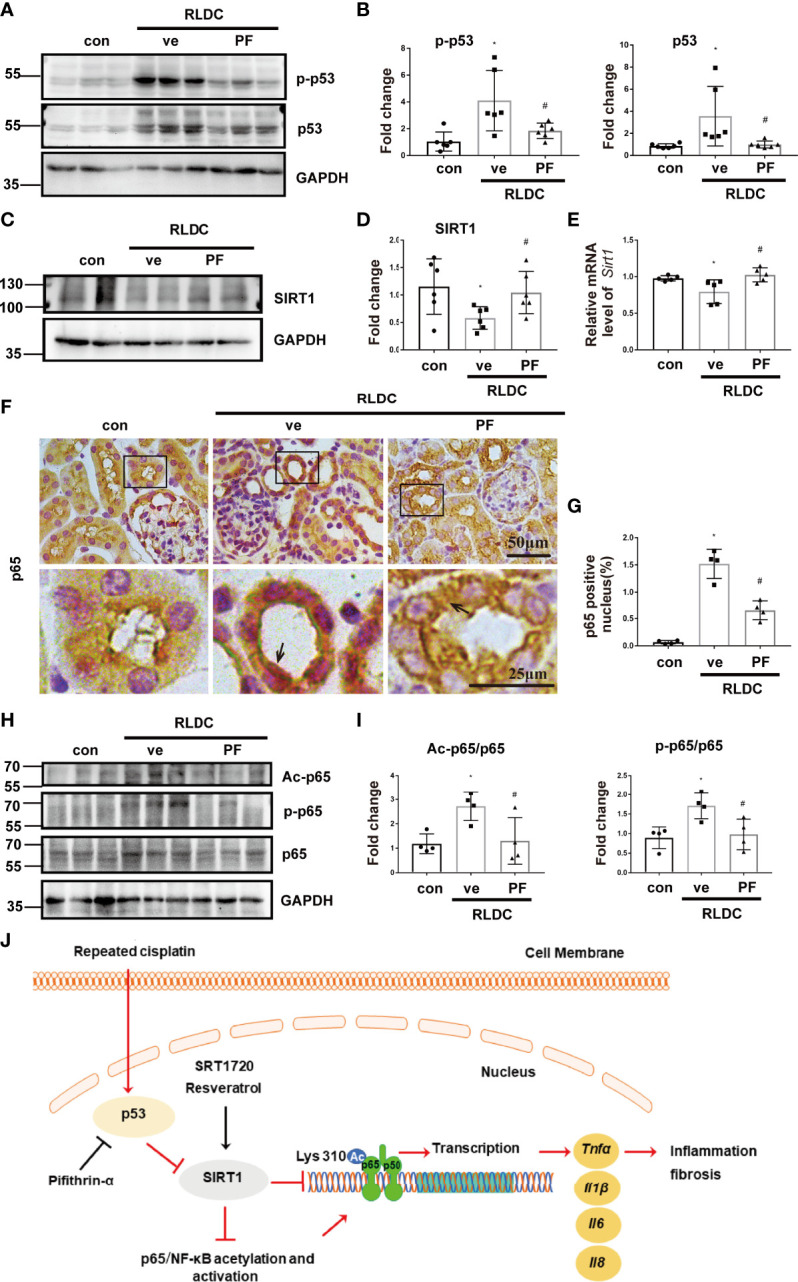
pifithrin-α prevents SIRT1 down-regulation and p65/NF-κB activation in post-RLDC mouse kidneys. Male C57BL/6 mice were given 4 weekly injections of 8 mg/kg cisplatin. After the last cisplatin injection, 2.2mg/kg pifithrin-α (PF) or vehicle solution (ve) was injected daily for 1 week to collect samples for analysis. **(A)** Representative immunoblots of p-p53, p53 and GAPDH of total protein extracted from mouse kidney (n=5). **(B)** Densitometry of p-p53 and total p53 (n=6). For densitometry, the protein level of control group was arbitrarily set as 1, and the signals of other conditions were normalized with the control group to indicate their protein fold changes. **(C)** Representative immunoblots of SIRT1 and GAPDH of total protein extracted from mouse kidney (n=5). **(D)** Densitometry of SIRT1 (n=6). **(E)** Effect of PF on mRNA levels of Sirt1. Data were normalized to GAPDH and expressed as fold change compared to controls (n=5). **(F)** Representative images of immunohistochemical staining of p65. **(G)** Statistical analysis of the percentage of p65 nuclear entry area to the total area (n=4). **(H)** Representative immunoblots of Ac-p65, p-p65, p65 and GAPDH of total protein extracted from mouse kidney (n=5). **(I)** Statistical analysis of Ac-p65/p65 and p-p65/p65 (n=4). **(J)** Schematic diagram: p53/SIRT1/NF-κB signaling axis in renal inflammation and fibrosis during post-RLDC kidney repair. Under normal physiological conditions, SIRT1 represses NF-κB signaling by deacetylating its key component p65. Following RLDC treatment, p53 is activated to repress SIRT1, leading to p65/NF-κB acetylation and activation inducing the transcription of various pro-inflammatory cytokines for renal inflammation and fibrosis. By inhibiting p53, pifithrin-α may prevent SIRT1 down-regulation and NF-κB activation. As SIRT1 agonists, SRT1720 and resveratrol activate SIRT1, resulting in p65 deacetylation to suppress NF-κB and renal inflammation and fibrosis. Data are expressed as mean ± SEM. *P<0.05 *vs*. the control group (Con), #P < 0.05 *vs*. RLDC with vehicle solution group (RLDC + ve).

We focused on p53 to understand the mechanism of SIRT1 down-regulation in post-RLDC kidneys mainly based on two facts: 1) RLDC induced a significant decrease in SIRT1 mRNA in BUMPT cells indicating SIRT1 down-regulation at the transcription level (**Figure 7E**), and p53 has been reported to regulate SIRT1 transcription (46); and 2) p53 is known to mediate cisplatin-induced AKI (47, 48), and we detected p53 activation in post-RLDC mouse kidneys (**Figure 8E**). We examined the effect of pifithrin-α (PF), a commonly used pharmacologic inhibitor of p53. As shown in **Figures 7A, B**, PF reduced the increase of p53 and p-p53 in RLDC-treated cells. Importantly, PF restored SIRT1 expression in these cells at both protein (**Figures 7C, D**) and mRNA (**Figure 7E**) levels. Bioinformatics analysis using JASPAR database (http://jaspar.genereg.net/) identified a potential p53 binding site (**Figure 7F**) on ​​the promoter of *Sirt1* gene. ChIP analysis showed that RLDC specifically increased the binding of p53 to the *Sirtl* gene promoter at this site (**Figure 7G**). As for the SIRT1/NF-κB pathway, inhibition of p53 with PF also reduced p65 phosphorylation and acetylation (**Figures 7H, I**). For further validation, we also knocked down p53 in mouse renal tubular cells (**Supplementary Figure 5A**) and found that the results were consistent with pharmacological inhibitors, i.e., p53 knockdown alleviated RLDC-induced SIRT1 reduction and p65 activation (**Supplementary Figures 5B–E**).

In mice, PF also inhibited p53 in post-RLDC kidneys and reduced SIRT1 down-regulation and p65/NF-κB activation including phosphorylation and acetylation (**Figure 8**). Collectively, these results indicate that p53 mediates SIRT1 down-regulation during post-RLDC kidney repair by directly repressing *Sirt1* gene transcription.

## Discussion

RLDC regimen is a common way to reduce the risk of nephrotoxicity in cisplatin chemotherapy, but, even with extreme clinical cautions, the incidence of acute and chronic kidney problems remains significant. Recent studies have verified the chronic effects of RLDC on kidneys in animal models; however, the underlying mechanism is largely unclear. There are three major findings in our current study as we have shown in **Figure 8J**: 1. RLDC induces aberrant NF-κB activation in renal tubular cells that contributes to chronic kidney injury by triggering the production of pro-inflammatory cytokines for a persistent inflammatory response; 2. SIRT1 is down-regulated in post-RLDC kidneys and this down-regulation contributes to NF-κB activation and associated chronic inflammation and kidney injury; 3. p53 mediates the down-regulation of SIRT1 by repressing its gene transcription in post-RLDC kidneys. Together, these results unveil the p53/SIRT1/NF-κB signaling axis in chronic renal inflammation and pathology during maladaptive kidney repair, highlighting potential therapeutic targets.

NF-κB is a transcription factor that plays important roles in the regulation of inflammation, cell death, survival, proliferation and differentiation (49, 50). In kidneys, NF-κB promotes inflammation by inducing the expression of pro-inflammatory cytokines in multiple renal disease settings (27). Inhibition of NF-κB in acute nephrotoxicity ameliorates kidney injury and tubular necrosis by attenuating inflammation (51), but the role of NF-κB in maladaptive kidney repair after AKI is less unclear. In this study, we demonstrate for the first time the continuous activation of NF-κB after RLDC treatment, accompanied by chronic inflammation, renal tubular damage and atrophy, and interstitial fibrosis. Inhibition of NF-κB resulted in a decrease in the expression of pro-inflammatory factors in tubule cells, less tubular damage and less fibrosis, supporting a pivotal role of NF-κB in maladaptive kidney repair following RLDC treatment.

Emerging studies have suggested a protective role of SIRT1 in the pathogenesis of renal fibrosis. In UUO, SIRT1 expression or activation in renal medullary interstitial cells attenuated the expression of COX2 and the subsequent renal fibrogenesis, linking SIRT1 in renal interstitial cells to oxidative stress signaling and renal fibrosis (36). In addition, SIRT1 activation could inhibit the TGFβ/Smad3 pathway in 5/6 nephrectomy and obstructed kidneys through epigenetic regulation of Smad3, leading to the amelioration of kidney fibrosis (35, 52). Nevertheless, it is known that, following injury, renal tubular cells secrete pro-inflammatory and pro-fibrotic cytokines for maladaptive kidney repair including fibrosis. Accordingly, SIRT1 may ameliorate these responses to improve kidney repair. Our current study provides insights into this mechanism. We show that SIRT1 can deacetylate p65 (a critical component of NF-κB) to prevent NF-κB activation in renal tubular cells. During kidney repair after RLDC treatment, SIRT1 was dramatically down-regulated, resulting in increased p65 acetylation and NF-κB activation leading to the production of various pro-inflammatory cytokines for maladaptive kidney repair. It is worth noting that SIRT1 may deacetylate other target proteins as well. For example, SIRT1 may alleviate cisplatin-induced renal senescence by deacetylating p53 (53). Future research needs to delineate the possible crosstalk between NF-κB signaling and other pathways in terms of SIRT1 regulation during maladaptive kidney repair.

To understand the mechanism of SIRT1 down-regulation following RLDC treatment, we focused on p53, which was implicated in transcriptional regulation of SIRT1 previously (46). In our study, RLDC induced p53 activation and the decrease of SIRT1 transcription in BUMPT cells and mouse kidneys (**Figures 7**, **8**). Moreover, inhibition of p53 with pifithrin-α prevented SIRT1 down-regulation (**Figures 7**, **8**). ChIP analysis further showed that RLDC specifically increased the binding of p53 to the promoter of the Sirt1 gene (**Figure 7G**), indicating a direct regulation of SIRT1 by p53. Pifithrin-α also reduced the phosphorylation and acetylation of p65 after RLDC treatment (**Figures 7H** and **8H**). All these results support a role of p53 in SIRT1 down-regulation during maladaptive kidney repair after RLDC treatment. Interestingly, SIRT1 may directly deacetylate p53 (53), suggesting a feedback mechanism to control SIRT1 expression.

The current study also suggests therapeutic potentials by targeting the p53/SIRT1/NF-κB signaling axis. First, considering the role of inflammation in the maladaptive repair process, inhibiting inflammation is a logical strategy for improving kidney repair. In this regard, Ravichandran et al. showed that depletion of CD4 T cells did not protect against low-dose cisplatin-induced AKI or subsequent renal fibrosis in mice with cancer, but it led to a weakened anti-cancer effect of cisplatin (17). In clinical trials, monoclonal antibodies that neutralize inflammatory factors have been used to treat kidney diseases. For example, CCR2 antagonists were used in the treatment of type 2 diabetes in a phase I clinical trial (54, 55). However, maladaptive kidney repair likely involves multiple inflammatory factors, and the key pathogenic inflammatory factors remain to be identified by experimental and clinical studies. In this regard, targeting of NF-κB, the “master” transcription factor for a myriad of pro-inflammatory cytokines, would shut down overall inflammation to prevent maladaptive kidney repair and facilitate normal kidney repair or functional recovery. Notably, the current study has unveiled SIRT1 as a negative regulator of NF-κB and pharmacological activation of SIRT1 resulted in deacetylation of p65 and suppression of NF-κB, which were associated with reduced renal inflammation and improved kidney repair. These observations support the therapeutic potential by activating SIRT1 and/or inhibiting NF-κB.

## Data Availability Statement

The original contributions presented in the study are included in the article/**Supplementary Material**. Further inquiries can be directed to the corresponding author.

## Ethics Statement

The animal study was reviewed and approved by the Animal Ethics Committee of the Second Xiangya Hospital of Central South University.

## Author Contributions

ZD and YF designed this study; YF performed most of the experiments; YF, YW, YL, and CT contributed to data analysis; ZD and YF analyzed results; GC and JC provided suggestions for manuscript preparation; ZD and YF contributed to manuscript writing. All authors contributed to the article and approved the submitted version.

## Funding

This study was financially supported by National Key R&D Program of China (2020YFC2005000), National Natural Science Foundation of China (82090024, 81720108008), the Natural Science Foundation of Hunan Province (2020JJ5798) and the Fundamental Research Funds for the Central Universities of Central South University (2021zzts0392).

## Conflict of Interest

The authors declare that the research was conducted in the absence of any commercial or financial relationships that could be construed as a potential conflict of interest.

## Publisher’s Note

All claims expressed in this article are solely those of the authors and do not necessarily represent those of their affiliated organizations, or those of the publisher, the editors and the reviewers. Any product that may be evaluated in this article, or claim that may be made by its manufacturer, is not guaranteed or endorsed by the publisher.
