# Supplementary material for: p53/sirtuin 1/NF-κB Signaling Axis in Chronic Inflammation and Maladaptive Kidney Repair After Cisplatin Nephrotoxicity
Source: Front Immunol. 2022 Jul 7;13:925738. doi: 10.3389/fimmu.2022.925738 (PMC9301469; doi:10.3389/fimmu.2022.925738)
Supplement: Supplementary file 1 [file DataSheet_1.pdf]

Supplementary Material

1 Supplementary Data

Supplemental Figure 1. RLDC treatment induces chronic kidney inflammation.

Supplemental Figure 2. Effects of SRT1720 on renal atrophy, kidney damage and renal function in post-RLDC mice.

Supplemental Figure 3. SRT1720 reduces protein expression of inflammatory factors following RLDC treatment.

Supplemental Figure 4. SRT1720 improves tubular proliferation and decreases tubular senescence in RLDC-treated mice.

Supplemental Figure 5. Knockdown of Trp53 increases RLDC-induced SIRT1 depletion and p65 activation.

2 Supplementary Figures

2.1 Supplementary Figure 1

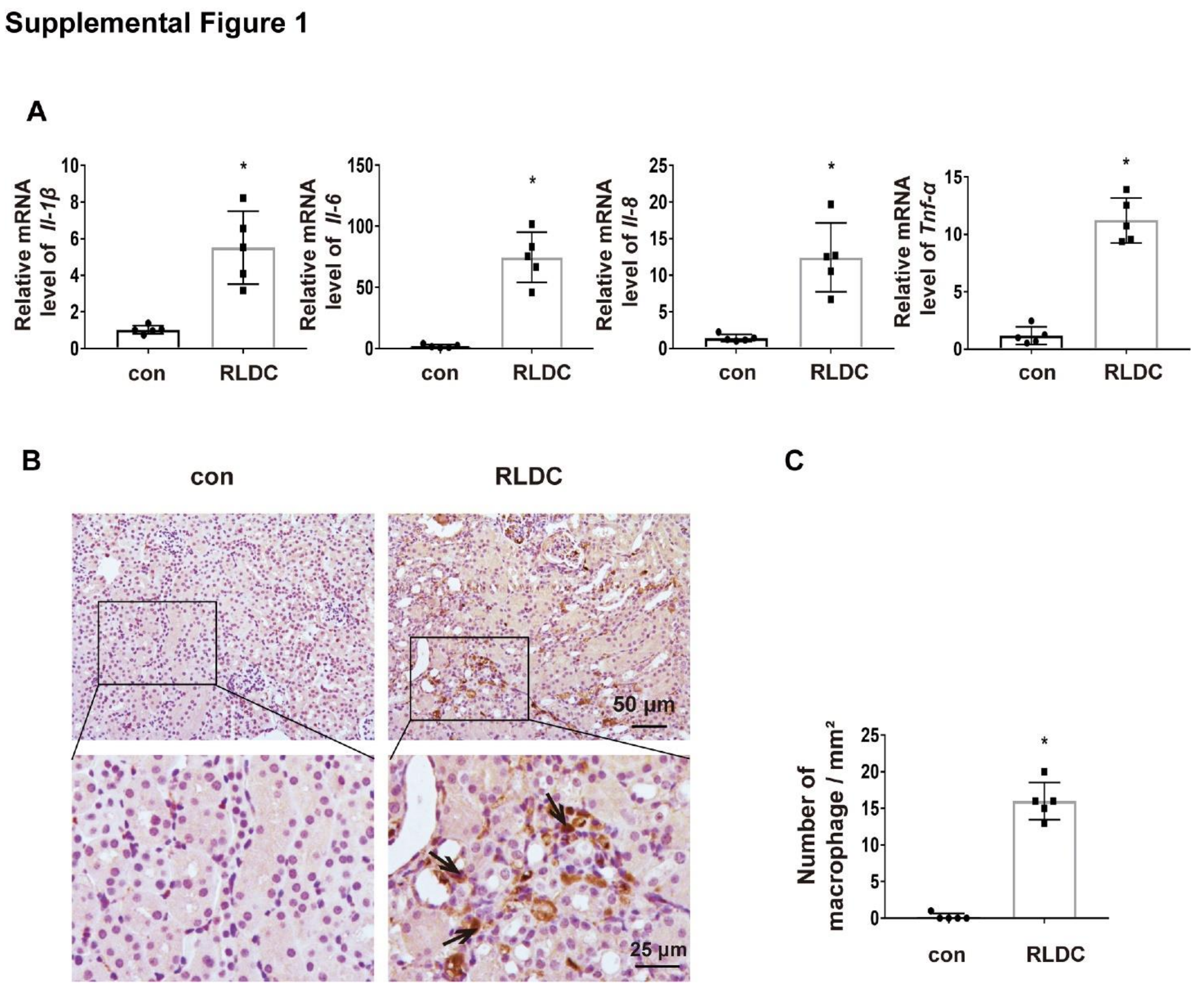

Supplementary Figure 1. RLDC treatment induces chronic kidney inflammation. Male C57BL/6 mice were injected weekly with 8 mg/kg cisplatin for 4 weeks to collect samples 1 month later. (A) mRNA levels of pro-inflammatory cytokines (*Il-1β, Il-6, Il-8, Tnf-α*) in kidney tissues quantified by qRT-PCR (n=5). The values were normalized to GAPDH and expressed as fold change compared to control (Con). (B) Representative images of immunohistochemical staining of F4/80 to show macrophage infiltration in kidneys. (n=5). (C) Counting of macrophages per square millimeter. Data are expressed as mean ± SEM. *P＜0.05 vs. the control group (Con).

2.2 Supplementary Figure 2

Supplemental Figure 2

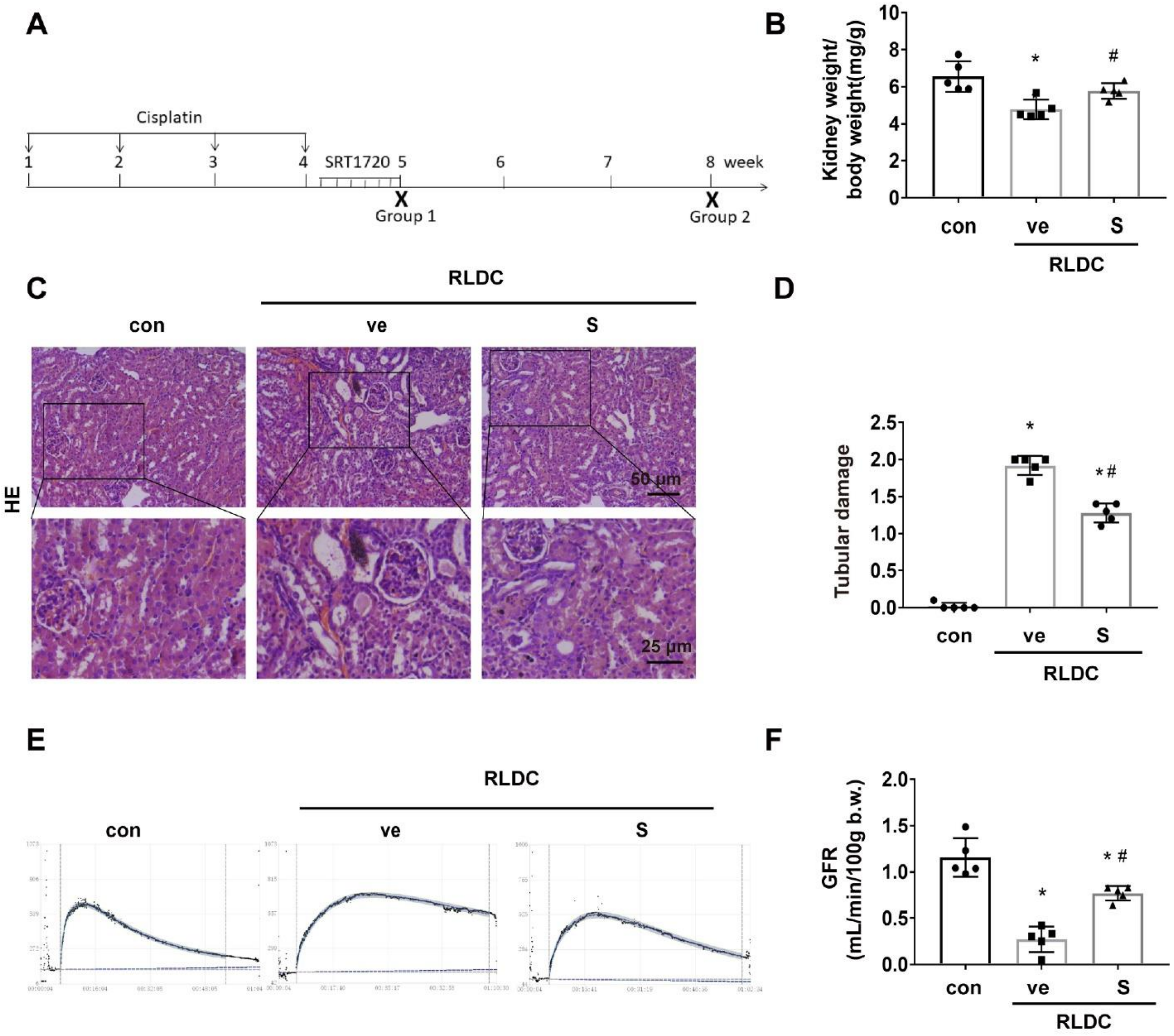

Supplementary Figure 2. Effects of SRT1720 on renal atrophy, kidney damage and renal function in post-RLDC mice. Mice were given 4 weekly injections of 8 mg/kg cisplatin (RLDC). Then, SRT1720 was injected into mice daily for one week. One group of animals were sacrificed immediately after SRT1720 treatment to examine the effects of SRT1720 on renal tubule damage, SIRT1 and p65 acetylation, while the other group of animals were terminated 3 weeks later to examine the long-term effects of SRT1720 on chronic renal inflammation and interstitial fibrosis. (A) Time-line of mouse treatment. (B) SRT1720 partially prevented kidney weight loss in post-RLDC mice. (n=5) (C) Representative images of HE staining showing the beneficial effect of SRT1720 in post-RLDC mice. (n=5). (D) Tubular damage score. (E) Representative tracings of GFR measurement by monitoring transcutaneous FITC-sinistrin clearance (n=5). (F) Quantitative analysis of GFR. Data are expressed as mean ± SEM. *P＜0.05 vs. the control group (Con), #P < 0.05 vs. RLDC with vehicle solution group (RLDC + ve).

2.3 Supplementary Figure 3

Supplemental Figure 3

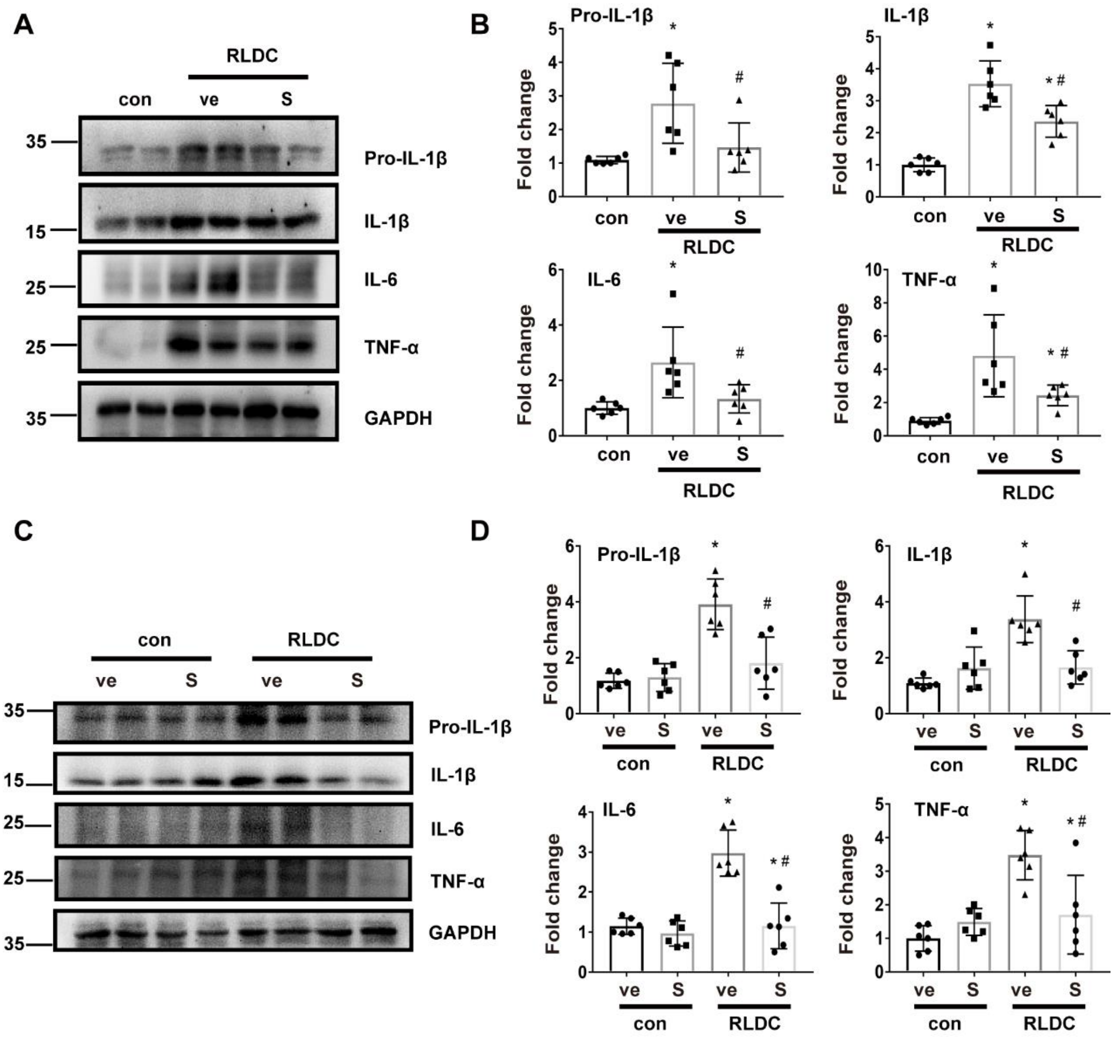

Supplementary Figure 3. SRT1720 reduces protein expression of inflammatory factors following RLDC treatment. (A-B) Mice were given 4 weekly injections of 8 mg/kg cisplatin (RLDC). Then, SRT1720 was injected into mice daily for one week. Animals were terminated 3 weeks later to examine the protein level of inflammatory factors. (A) Representative immunoblots of pro-IL-1β, IL-1β, IL-6, TNF-α and GAPDH in RLDC (8w) mice (n=6). (B) Densitometry of pro-IL-1β, IL-1β, IL-6, TNF-α. The protein level of control group was arbitrarily set as 1, and the signals of other conditions were normalized with the control group to indicate their protein fold changes. (C-D) BUMPT cells were incubated with 2μm cisplatin for 7h each day for 4 days, and then treated with 2.5μM SRT1720 (S), or vehicle solution (ve) for 17h in cisplatin-free medium. (C) Representative immunoblots of pro-IL-1β, IL-1β, IL-6, TNF-α and GAPDH in RLDC cells (n=6). (D) Densitometry of pro-IL-1β, IL-1β, IL-6, and TNF-α. Data are expressed as mean ± SEM. *P< 0.05 vs. the control group (Con or Con + ve), #P < 0.05 vs. RLDC with vehicle solution group (RLDC + ve).

Supplementary figure 4

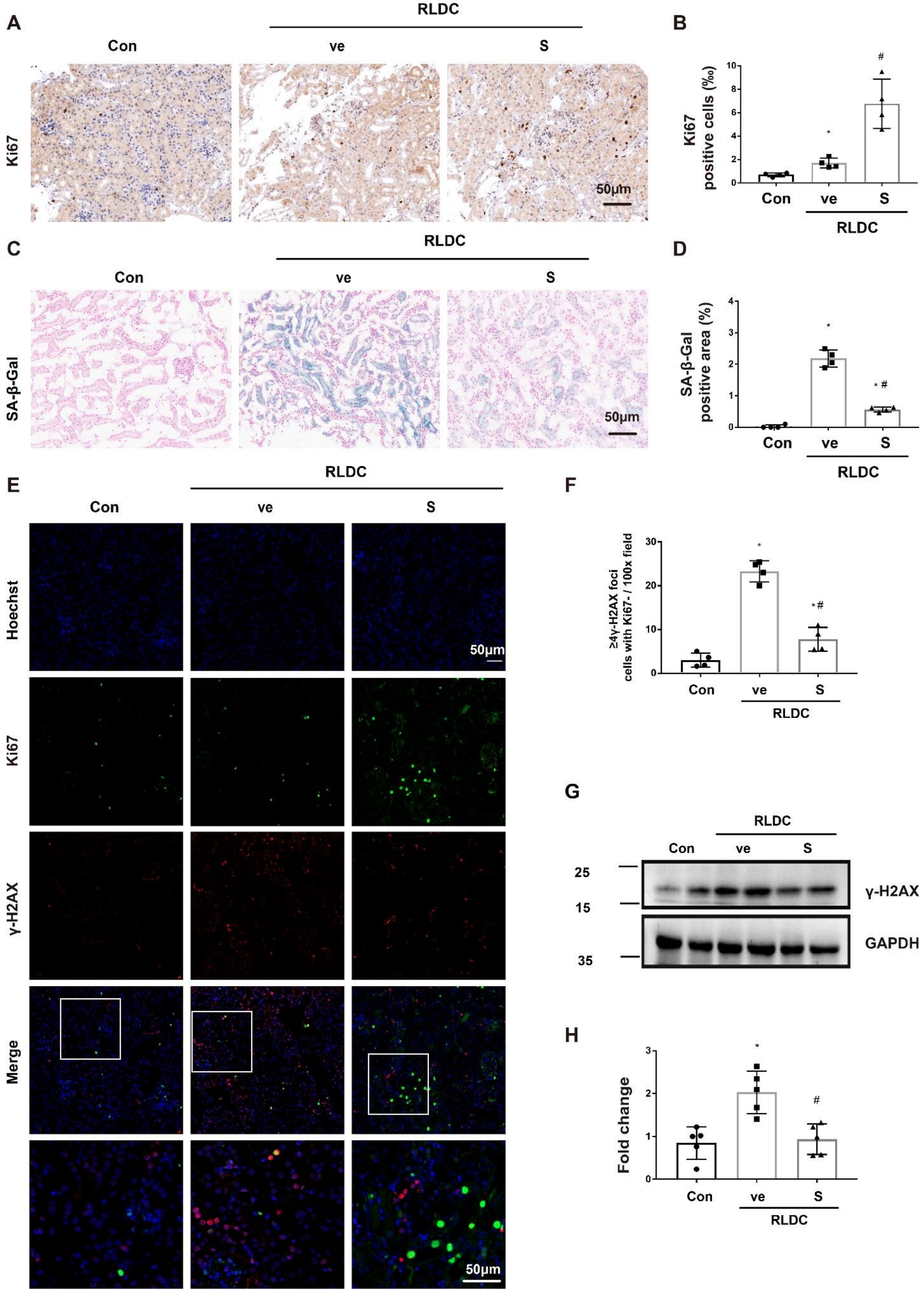

Supplementary Figure 4. SRT1720 improves tubular proliferation and decreases tubular senescence in RLDC-treated mice. Male C57BL/6 mice were given 4 weekly injections of 8 mg/kg cisplatin. After the last cisplatin injection, 100mg/kg SRT1720 (S) or vehicle solution (ve) was injected daily for 1 week and then the mice were kept for another 3 weeks to collect samples for analysis. (A) Representative images of Ki67 immunohistochemistry. (B) Quantitative analysis of Ki67 positive cells. (C) Representative images of SA-β-Gal staining. (D) Quantitative analysis of SA-β-Gal positive area. (E) Co-immunostaining of Ki67 (green) and γ-H2AX (red). Tubular cells with more than four γ-H2AX foci in nuclei and negative for Ki67 staining were considered senescent. (F) Quantification of the Ki67 negative cells with four or more γ-H2AX foci in kidney tubules. (G) Representative immunoblots of γ-H2AX and GAPDH. (H) Densitometric analysis of the immunoblots of γ-H2AX. The protein level of control group was arbitrarily set as 1, and the signals of other conditions were normalized with the control group to indicate their protein fold changes. Data are expressed as mean ± SEM. N ⩾ 4. *P<0.05 vs. the control group (con), #P < 0.05 vs. vehicle solution (ve) plus RLDC group.

Supplementary figure 5

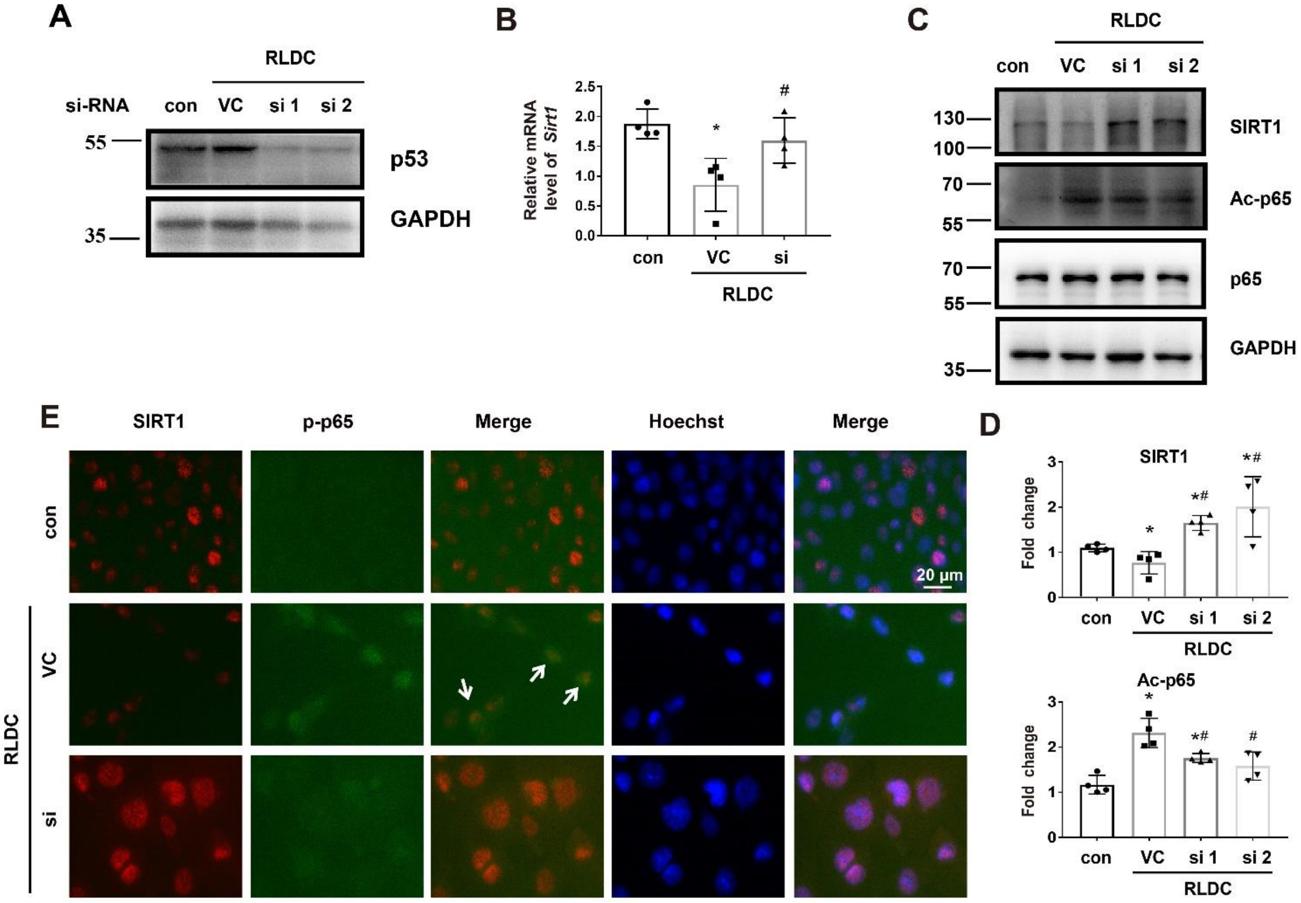

Supplemental Figure 5. Knockdown of Trp53 increases RLDC-induced SIRT1 depletion and p65 activation. BUMPT cells were transfected with 50nM *Trp53* siRNA1 (si1), siRNA2 (si2), or control siRNA (VC), and then subjected to 4-day basic RLDC treatment. (A) Representative immunoblots of p53 and GAPDH (loading control) after transfection. (B) Quantitative analysis of the mRNA level of *Sirt1* after *Trp53* knockdown (n=4). Data were normalized to *Gapdh* and expressed as fold change over

controls. (C) Representative immunoblots of SIRT1, Ac-p65, p65 and GAPDH (loading control) of total protein extracted from cells (n=4). (D) Quantitative analysis of the immunoblots of SIRT1 and Ac-p65/p65. (E) Immunofluorescence co-staining of SIRT1 and p-p65 after si-*Trp53* transfection. Arrows point to decreased nuclear SIRT1 expression and increased p-p65 expression after RLDC. Data are expressed as mean ± SEM. *$P < 0.05$ vs. the control group (con), #$P < 0.05$ vs. VC siRNA plus RLDC group.
